# Augmented Reality in Outpatient Care: A Narrative Review

**DOI:** 10.1049/htl2.70025

**Published:** 2025-11-22

**Authors:** Archan Khandekar, Aryan Shah, Luis A. Esparza Miranda, Frida Toscano Bello, Bruno Liebl, Jonathan Ryan, Pedro Angelo Basei de Paula, Ansh Bhatia, Timothy Guerard, Dipen J. Parekh

**Affiliations:** ^1^ Desai Sethi Urology Institute University of Miami Miller School of Medicine Miami Florida USA; ^2^ Federal University of Paraná (UFPR) Curitiba Brazil; ^3^ Dr. Kiran C. Patel College of Allopathic Medicine (NSU MD) Davie Florida USA; ^4^ Department of Internal Medicine Federal University of Paraná Curitiba Brazil; ^5^ Department of Interventional Radiology University of Miami Miller School of Medicine Miami Florida USA

**Keywords:** augmented reality, healthcare technology, outpatient care, patient education, patient understanding, procedural guidance, rehabilitation, remote monitoring

## Abstract

**Introduction:**

Augmented reality (AR) is seeing an increase in its applications in healthcare, but its reach in outpatient care remains undefined. Patients in outpatient settings face poor medical understanding. AR may help address this gap between patients and physicians through immersive and interactive models and supporting tools. This narrative review aims to evaluate the status of AR in outpatient care, categorise its applications, and identify limitations and future research needs.

**Methods:**

Four databases–PubMed, Embase, Web of Science and Cochrane Library–were conducted for peer‐reviewed studies published from January 2015 to February 2025. Studies were included if they regarded AR interventions in outpatient care settings. Studies were analysed and grouped thematically into five clinical domains of AR intervention.

**Results:**

After review, 19 studies–spanning 987 participants–were included. AR applications were categorised into patient education and engagement (n = 3), cognitive and functional assessment (n = 3), device interaction and remote monitoring (n = 3), procedural guidance in outpatient interventions (n = 5), and rehabilitation and functional recovery support (n = 5). Most included studies were pilot studies (n = 6) and had relatively small sample sizes (median = 28). Studies proved that AR interventions consistently improved patient understanding, engagement and procedural support. Nevertheless, studies faced limitations including the need for specialised and bulky hardware–which affected patient comfort as well–reliability issues, technical difficulties and platform‐specific inconsistencies.

**Conclusion:**

AR has been proved to have the potential to improve outpatient care across five main areas: patient education, cognitive and functional assessment, medical device interaction, procedural guidance and rehabilitation. Studies consistently support that AR enhances patient comprehension, engagement and procedural accuracy while allowing for remote monitoring and personalised therapy. Furthermore, AR interventions demonstrate high usability and clinical relevance. Nevertheless, limitations such as hardware complexity and inconsistent technical performance remain. Future research should prioritise large‐scale RCTs and strategies to integrate AR into pre‐existing digital workflows.

## Graphical abstract

Augmented Reality (AR) is increasingly used in healthcare, but its role in outpatient care is not well defined. This review of 19 studies found that AR, in outpatient settings, assists in patient education and engagement, cognitive and functional assessment, remote monitoring, outpatient procedures and rehablilitation. Future research should focus on integrating AR into clinical workflows and conducting larger‐scale trials to address hardware and reliability challenges.

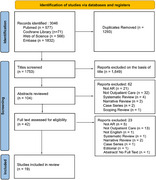

## 1 Introduction

Augmented reality (AR) is a technology that overlays information–such as text, images and models–onto the user's real‐world view in real time.[1] This experience is typically delivered through smartphones, tablets, head‐mounted displays and complex hardware systems. Although AR has been in the works since 1968, with applications in defence and spacecraft navigation, AR did not gain momentum until Tom Caudell coined the term ‘augmented reality’ in 1992 [2, 3]. In clinical surgery, AR was first used in the early 2000s for spinal procedures in Japan. More recently, in 2020, neurosurgeons at Johns Hopkins utilised advanced AR headsets during spinal fusion surgery, marking a significant step toward mainstream intraoperative use [4]. Since then, there has been an increasing number of applications of AR amongst different specialities in medicine.

Outpatient care–clinic visits, imaging services, rehabilitation and some surgical and medical procedures–plays an important role in ensuring a patient's well‐being. Also, patients receive medical and self‐care instructions from physicians in out‐patient settings. There is a trend of increased outpatient care utilisation with a 31% increase in the number of outpatient visits in the United States from the year 2000 to 2023 [5].

Unfortunately however, outpatient care often suffers from communication gaps and the lack of medical understanding in patients [6]. Patients frequently leave outpatient appointments with confusion about their diagnosis, medications, or any other instructions, leading to poor adherence, hospital readmissions, and suboptimal health outcomes [6]. As such, an increase in medical understanding can significantly improve patient adherence rates [7]. AR can potentially breach this gap between physician and patient and play an important role in improving patient understanding about their diagnoses and treatment plans [8]. There are multiple publications in different sub‐specialities focusing on applications of AR in various aspects of outpatient care. Similarly, there is no review or meta‐analysis published on the role of AR in an outpatient setting.

Hence, we conducted a narrative review focusing on applications of AR in outpatient care. We believe that our study will provide a birds‐eye view of the current status of AR in outpatient care and will stimulate further applications to improve patient comprehension of their medical condition and treatments. This in turn would improve the efficiency of medical care, leading to improved patient satisfaction and outcomes.

## 2 Methods

This review was conducted and reported in accordance with the Preferred Reporting Items for Systematic Reviews and Meta‐Analyses (PRISMA) guidelines. The review protocol was registered with the PROSPERO international prospective register of systematic reviews (registration number: CRD420250648110). Institutional Review Board approval was not required, as the study involved no direct patient data.

**FIGURE 1:**
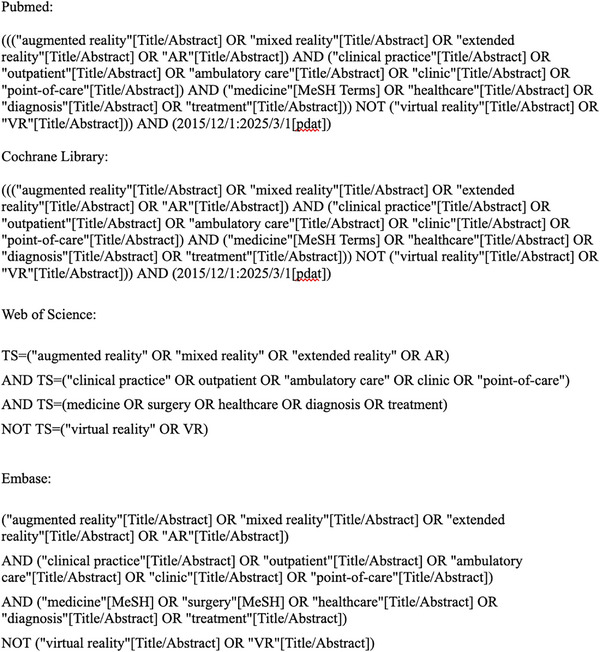
Complete search strategies for PubMed, Web of Science, Cochrane, and Embase databases.

A search strategy was designed to identify peer‐reviewed publications reporting on the use of augmented reality (AR) in outpatient care. Four databases were searched: PubMed, Cochrane Library, Embase and Web of Science, covering studies published between 1 January, 2015, and 1 February, 2025. Search terms included a combination of keywords and Boolean operators such as: ‘augmented reality’, ‘mixed reality’, ‘extended reality’, ‘AR’, ‘clinical practice’, ‘outpatient’, ‘ambulatory care’, ‘clinic’, ‘point‐of‐care’, ‘medicine’, ‘surgery’, ‘healthcare’, ‘diagnoses’ and ‘treatment’. The complete search strategy is shown in Figure 1.

**FIGURE 2:**
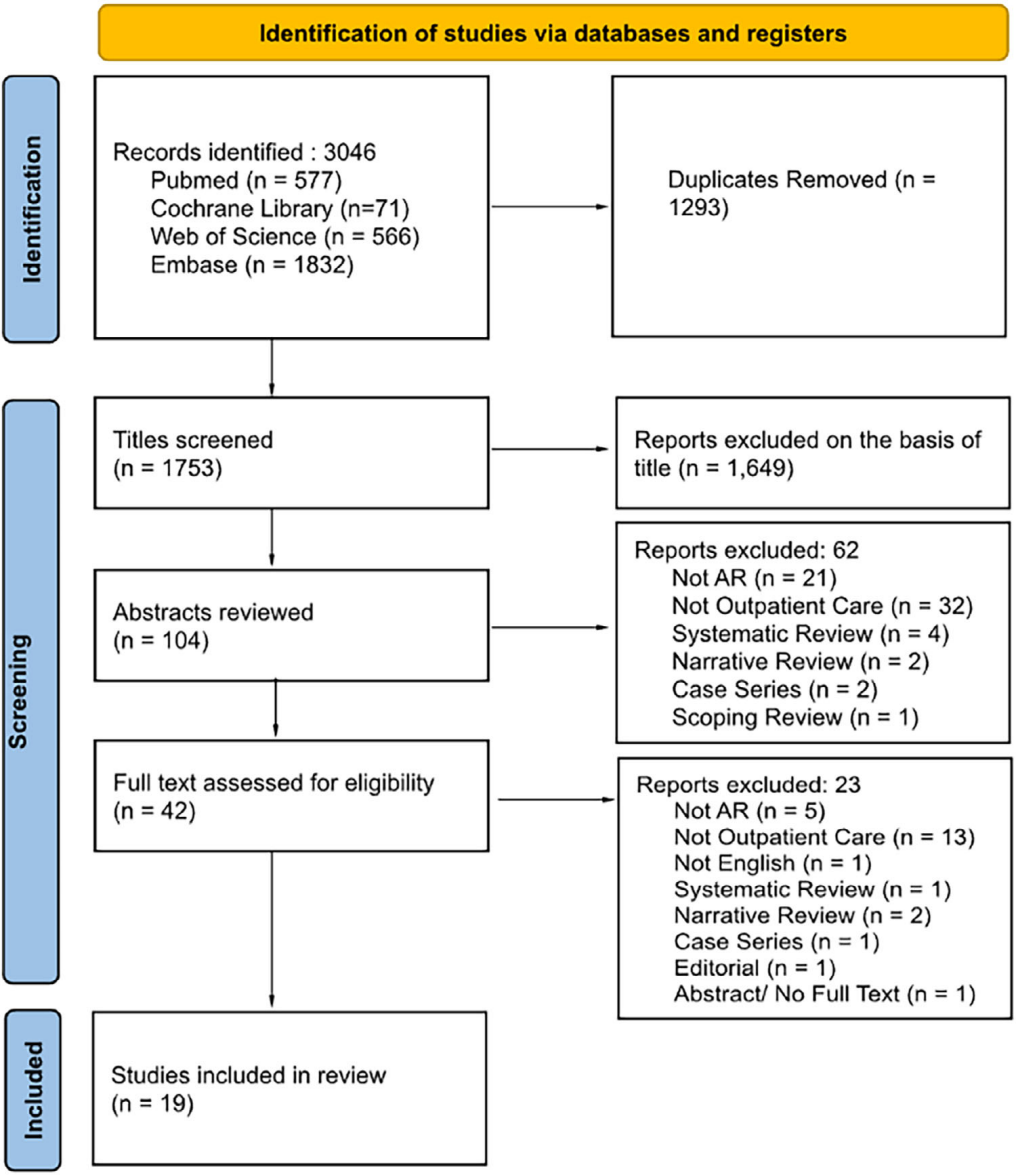
PRISMA flow chart describing the process of article selection.

All retrieved articles were screened in two stages. First, titles and abstracts were independently reviewed by three authors (AS, LE and FT). Articles were excluded if they were duplicates, reviews, or lacked relevant information on AR applications in outpatient care. Out of three authors, there was a discrepancy, as one of those three did not agree initially regarding the exclusion of five abstracts and two full text articles. This discrepancy was resolved through discussion and consensus among the reviewers in consultation with another author (AK). In the second stage, 43 full‐text articles were independently assessed for eligibility by the same reviewers. Ultimately, 19 studies met all inclusion criteria and were selected for final analysis. The study selection process is illustrated in the PRISMA flow diagram (Figure 2).

Data extraction was performed independently by two reviewers (AS and LE) using a predefined and piloted extraction form in Microsoft Excel. Extracted data included study design, sample size, country of origin, type of AR intervention, thematic category of use and reported outcomes. The extracted data were cross‐verified to ensure consistency and accuracy. Any disagreements were resolved by discussion among the review team.

We subdivided AR applications in outpatient care into the following five broad subtypes based on their applications in outpatient workflow.

1. AR‐enhanced cognitive and functional assessment
2. AR‐based patient education and engagement
3. AR‐assisted guidance and interventions in outpatient procedures
4. Rehabilitation and functional recovery support
5. AR‐mediated device interaction and remote monitoring

## 3 Results

A comprehensive evaluation included a total study population of 987 participants, extracted from 19 studies that met the inclusion criteria, out of an initial pool of 3,046 records screened. The study selection process is detailed in the PRISMA flow diagram (Figure 2).

The included studies encompassed a variety of methodological designs and AR intervention themes. Specifically, included studies were pilot studies (n = 6), employed prospective observational designs (n = 4), randomised control trials (RCTs) or protocol‐based RCTs (n = 3), a cross‐sectional studies (n = 2), a comparative study (n = 1), a cohort study (n = 1), a proof‐of concept study (n = 1) and an experimental prototype evaluation (n = 1).

Included studies spanned 10 countries: the United States (n = 5), China (n = 3), the Netherlands (n = 3), South Korea (n = 2), India (n = 1), Germany (n = 1), the United Kingdom (n = 1), Italy (n = 1), Spain (n = 1) and Sweden (n = 1).

The augmented reality (AR) interventions investigated were classified into five key thematic areas: patient education and engagement (n = 3), cognitive and functional assessment (n = 3), device interaction and remote monitoring (n = 3), procedural guidance in outpatient interventions (n = 5) and rehabilitation and functional recovery support (n = 5).

Out of the 19 studies included, 14 explicitly mentioned participants giving informed consent [9, 10, 11, 12, 13, 14, 15, 16, 17, 18, 19, 20, 21, 22]. The remaining five studies–consisting of a prototype study (n = 1), pilot studies (n = 3) and a prospective study (n = 1)–did not explicitly mention informed consent from patients [8, 23, 24, 25, 26].

### 3.1 AR‐Based Patient Education and Engagement

In ophthalmology, an Eye MG AR module used smartphones and printed templates to deliver marker‐based AR counselling for glaucoma. There was a statistically significant increase in patients' understanding of glaucoma pathology and anatomy. However, there were limitations: iOS paywall access and the need for printed materials [9]. In oncology, a Microsoft HoloLens 2 was utilised with the HoloAnatomy platform to deliver preoperative education for pancreatoduodenectomy patients. Results showed significant improvements in comprehension of surgical risks, procedural steps and recovery (Z = −2.524, *p* = 0.012) [10]. In neurology, HoloLens‐2‐based stroke education outperformed standard tele‐stroke methods on all patient‐rated metrics of satisfaction and immersion (median score: 48 vs. 32, *p* = 0.012A) [11].

### 3.2 AR‐Enhanced Cognitive and Functional Assessment

In 2023, the AR‐based Altoids app outperformed standard tests in identifying preclinical and prodromal Alzheimer's; furthermore, there were moderate correlations with in‐clinic assessments and feasibility of at‐home use. However, limitations included poor test‐retest reliability–especially on iOS– device variability, inconsistent app versions and potential cognitive bias in unsupervised environments [12]. In 2024, a HOLOBalance AR‐based telerehabilitation system was compared with standard care. The AR group showed greater improvement in functional gait and balance scores, with 69% recommending the system; however, 27% experienced technical issues such as connectivity and sensor malfunctions [13]. In 2025, a Microsoft HoloLens 2 was utilised to capture eye, hand and voice metrics from a healthy subject during standardised neurological tasks. The system successfully tracked tremor frequency, tap timing, turn angle, vocal characteristics and reaction time without notable limitations [23].

### 3.3 AR‐Mediated Device Interaction and Remote Monitoring

In 2016, an android app used the Vuforia SDK to automate peak expiratory flow rate measurements via AR recognition of custom stickers on peak flow meters; the system achieved 96% accuracy within ± 20 L/min of manual readings with high usability and low training requirements. However, drawbacks noted by authors included camera autofocus delays, sensitivity to angle and placement and performance degradation at high flow rates [14]. In 2019, mobile AR prototypes displaying metadata for clinical equipment were tested, and results showed an excellent usability score (SUS = 87), with users–clinicians and biomedical engineers–reporting improved interaction and understanding of unfamiliar devices without notable limitations [15]. In 2024, the Magic Leap 2 and Microsoft HoloLens 2 headsets–with integrated gamified gait‐and‐balance exercises with real time feedback and remote clinician monitoring–were evaluated for their feasibility. While outcome data were not yet reported, early observations cited device weight, tethering and the need for initial setup as main limitations, though overall feasibility was established [24].

### 3.4 AR‐assisted Guidance and Interventions in Outpatient Procedures

In 2020, an android AR app used facial recognition to guide Botox injections. Results demonstrated a mean landmark recognition error of 0.40 ± 0.25 mm for frontal views, with all angle errors ranging from 0 to 3.0 mm, thereby deeming AR acceptable for aesthetic procedures. However, the app lacked real‐time imaging and was limited to static visual guidance [16]. In 2024, the ARMedicalSketch for 3D interactions with medical images was introduced. It improved sketch accuracy and efficiency, reduced cognitive workload, and achieved higher usability scores than 2D alternatives. Still, it was a prototype and had not been validated for clinical work [25]. In 2022, the HoloPOCUS system utilised HoloLens 2 and Azure Kinect for ultrasound guidance in phantom biopsies. Results showed that AR significantly reduced procedure time and achieved sub‐millimetre tracking accuracy, with no notable limitations [26]. In 2023, Magic Leap 1 smart glasses were used in prostate biopsies to overlay MRI‐identified lesions in real time, showing a 90% targeting success rate, with users finding the system intuitive [17]. The same year, Microsoft HoloLens 2 was utilised for paediatric preoperative planning. AR reconstructions enhanced anatomical clarity, decreased planning time and supported safer procedural strategies. Although outcome data was lacking and dependent on preprocessed imaging [18].

### 3.5 AR‐Based Rehabilitation and Functional Recovery Support

In 2018, three customised AR games were utilised to assess upper limb motor control in Parkinson's disease and post‐stroke patients. The AR system–combining leap motion, Kinect and the AIRO II AR headset–captured speed, reach and goal‐directed movement. It was able to differentiate impairment types and scored well in usability, though limitations of field of view and ceiling/floor effects were noted [19]. In 2021, the UINCARE Home AR system was tested for adhesive capsulitis with 25‐joint real‐time tracking, exercise feedback and remote monitoring. However, outcome data was not reported [20]. In 2023, a smartphone‐based AR protocol was developed for meniscus injury rehab, targeting pain and function improvements [21]. The same year, remote musculoskeletal exams using AR were compared to in‐person exams. The remote system, ARTESH, showed strong inter‐rated agreement for PROM and MIS, though limitations included force range, specialised hardware and bandwidth needs [22]. In 2025, wearable AR and AR‐marked virtual limbs were utilised to reduce phantom limb pain. Pain decreased by 64.5‐68.2%, with over 70% of patients achieving clinically meaningful relief. The system proved effective but required therapist training [22].

## 4 Discussion

This narrative review on applications of AR in outpatient care demonstrates that AR technology is being actively implemented across diverse outpatient care applications. The concentration of studies in the United States (26.3%) and China (15.8%) reflects the current centres of AR development but raises questions about global applicability. The predominance of pilot studies (31.6%) and small sample sizes (median = 28) indicates that the field is still in early development stages.

Only three studies were randomised controlled trials (RCT)–with one being a pilot RCT– limiting our ability to draw causal conclusions about AR effectiveness [9, 12, 22]. The heterogeneity of outcome measures across studies prevented quantitative meta‐analysis, highlighting the need for standardised outcome metrics in future AR research.

Although AR proved to be useful across all categories, several consistent limitations emerged, as mentioned in Tables 1, 2, 3, 4, 5. First of all, for AR, there is a need for specialised equipment–including AR headsets–and high‐speed internet, both of which may be limitations to its implementation in outpatient care globally [10, 11, 13, 23, 24, 17, 18, 19, 22, 26, 27]. Additionally, there are platform‐specific performance variations and availability, especially on AR‐implementations based around smartphones, as there were differences between iOS versus android software [9, 12, 14]. Moreover, since AR systems can consist of various different hardware, there are connectivity and sensor reliability issues, and the multiple hardware may also lead to uncomfortable device weight and ergonomic issues [24]. AR systems also come with technical difficulties [12, 13, 14].

**TABLE 1:** AR‐based patient education and engagement.

Author, country, year, study type	AR intervention details	Sample size (n), population characteristics	Outcome measure	Key findings	Limitations
Ramesh et al. [8] India 2024 Perspective study with user‐srvey‐based analysis	Consumer smartphones used with marker‐based AR using printed template and camera‐based tracking.	100; details not given	Patient understanding of glaucoma anatomy and pathology. Effectiveness of AR‐based counselling in clinics	Statistically significant improvement in comprehension scores for all evaluated domains	The AR module on IOS is behind a paywall. AR template needs to be printed and used
Heard et al. [9] United States of America 2024 Randomised control pilot study	Microsoft HoloLens 2 used alongside HoloAnatomy platform for 3D anatomical procedural visualisation	19 Patients undergoing preoperative education for planned pancreatoduodenectomy	Patient understanding and comfort regarding their diagnoses and surgical procedures assessed via pre‐ and post‐intervention surgery (5 point Likert scale)	AR groups showed statistically significant improvements to overall comprehension (Z = ‐2.524, *p* = 0.012); Specifically in understanding surgical risks, operative steps and recovery	No major disruptions noted; need for specialised equipment–AR Headsets.
Weibel et al. [10] United States of America 2024 Prospective user study	HoloLens 2‐based mixed reality system for real‐time 2D/3D ultrasound guidance using stereo camera fiducial tracking and volume reconstruction	29 Non‐acute neurology clinic patients	Patient‐rated satisfaction and immersion (5‐point Likert scale); comparison between Tele‐stroke and Holo‐stroke	Holo‐stroke significantly outperformed tele‐stroke on all metrics (e.g., median score: 48 vs. 32, *p* < 0.00001); high immersion and clarity in image and provider interaction.	None noted; need for specialised equipment–AR Headsets.

**TABLE 2:** AR‐enhanced cognitive and functional assessment.

Author, country, year, study type	AR intervention details	Sample size (n), population characteristics	Outcome measure	Key findings	Limitations
Muurling et al. [11] The Netherlands 2023 Cross‐sectional study	AR‐based app (Altoids) to assess cognitive functions related to instrumental activities of daily living (IADLs) in early Alzheimer's disease, through spatial tasks like object placement and fire drill simulations on smartphones and tablets.	121 community‐dwelling adults aged >50 from 13 European study sites, with exclusions for conditions affecting cognition or daily functioning.	AR‐derived digital score Cognitive score (composite) A‐IADL questionnaire score Classification performance of AR versus standard tests Feasibility and reliability of home testing Test‐retest reliability Association with standard cognitive tests	The study found that the AR app effectively distinguished healthy controls from preclinical and prodromal AD participants, outperforming standard cognitive tests in detecting preclinical AD. At‐home testing was feasible, with moderate correlation to in‐clinic results, and no learning effects were observed.	Poor test‐retest reliability (esp. iOS); varied app versions; technical issues; unstandardised devices; potential cognitive bias at home.
Pavlou et al. [12] United Kingdoms 2024 Pilot RCT Study	AR headsets, inertial measurement unit sensors, and pressure‐measuring insoles with HOLOBalance system.	109 Older adults(median age: 73) at risk for falls	Feasibility, acceptability and safety; Functional gait assessment (FGA), mini‐balance evaluation systems test (mini‐BESTest), cognitive function (MoCA), fear of falling (FES‐I), disability(WHODAS 2.0), balance confidence (ABC scale)	76.15% completion rate; 69% of participants would recommend HOLOBalance; FGA and Mini‐BESTest scores showed more improvement in AR group compared to control.	Technical difficulties experienced by 27% of participants (connection issues, sensor problems); need for specialised equipment–AR Headsets.
Calame et al. [13] United States of America 2025 Proof‐of‐concept Pilot Study	Microsoft HoloLens 2 used for eye tracking, hand tracking, and voice analysis.	One healthy volunteer (non‐naïve, no diagnosed movement disorder)	Hand, eye and voice metrics during standardised neurological tasks: tremor frequency, tap amplitude/time, turn angle, reaction time, tortuosity, vocal pitch and jitter/shimmer.	All tasks were successfully captured and analysed using the headset. The movement and vocal features showed high fidelity tracking, like external metronome stimuli.	None noted other than the need for specialised equipment–HoloLens 2.

**TABLE 3:** AR‐mediated device interaction and remote monitoring.

Author, country, year, study type	AR intervention details	Sample size (n), population characteristics	Outcome measure	Key findings	Limitations
Chamberlain et al. [23] United States of America 2016 Pilot Study	Using an Android app developed with the Vuforia SDK, the system detected a custom‐printed sticker attached to a peak flow meter to measure PEFR, provided real‐time visual feedback, saved readings in CSV format, and allowed data export for analysis.	3 technical test: 50 readings; field test: 3 clinicians (1 doctor, 2 nurses)	Accuracy of automated AR‐based readings versus manual readings Usability metrics: task completion time, training requirements	The system showed 96% accuracy within acceptable error (<20 L/min) and strong agreement with manual readings. It required minimal training, was low‐cost, and avoided the need for electronics. Drawbacks included autofocus delays, camera angle sensitivity, and higher errors at high flow rates.	Camera autofocus delays, sticker placement affecting accuracy, minimal user training, errors at high PEFR values, the need for calibration and manufacturing variability.
Escalada‐Henrández et al. [14] Spain 2019 Pilot Study	Mobile AR application prototype for displaying detailed information about medical devices using image recognition	11 nurses, and 6 nurses for usability testing; 280 healthcare professionals	User‐perceived utility Usability and navigability Accuracy and clarity of information delivery	AR improved users' ability to interact with unfamiliar equipment; Described as ‘very intuitive’.	Prototype phase only; tested in a simulated environment; limited to a few types of medical equipment.
Hardeman et al. [15] The Netherlands 2024 Prospective clinical feasibility study	Tethered Magic Leap 2 or untethered Microsoft HoloLens 2 with real‐time feedback, gamified exercises and remote monitoring via web portal.	24 volunteers with Parkinson's disease (Hoehn and Yahr stages 2–4)	User experience, adherence, safety, Gait and balance improvement.	Not applicable	Device weight (HoloLens), tethering (Magic Leap), and technical learning curve; Need initial Wi‐Fi setup and AR environment configuration. Specs differ across AR glasses.need for specialised equipment–AR hardware.

**TABLE 4:** Procedural guidance and AR‐augmented interventions in outpatient procedures.

Author, country, year, study type	AR intervention details	Sample size (n), population characteristics	Outcome measure	Key findings	Limitations
Kim et al. [24] South Korea 2020 Pilot Study	Android‐based AR app using facial recognition (Dlib + OpenCV) to overlay Botox injection guides on patient faces.	28 Healthy adult volunteers, static simulation in a studio setting with controlled head positioning.	Accuracy of AR landmark recognition for Botox injection points	Mean error of 0.40 ± 0.25 mm for frontal views, with errors ranging from 0.0 to 3.0 mm across all angles. Accuracy varied by view, except for downward tilt, but was considered acceptable for Botox and filler procedures.	Use of standard model rather than patient‐specific anatomy No integration of real‐time medical imaging Not suitable for use as a navigation system; more of a visual guide
Zhang et al. [16] China 2024 Comparative Study	ARMedicalSketch, a novel augmented reality (AR) system designed for 3D sketching and interaction with medical images.	25; 23 biomedical engineering students (user study) 2 clinical experts (orthopedic and neurosurgeon)	Efficiency and accuracy in medical sketching tasks System usability (SUS) and workload (NASA‐TLX)	Improved sketching efficiency and accuracy for medical images, especially in complex 3D tasks. System Usability: Prototype 1 (ARMedicalSketch) scored higher in usability and satisfaction compared to Prototype 2 (traditional 2D system). Workload: Lower cognitive burden for users interacting with ARMedicalSketch	Small, non‐clinical sample; prototype stage only; not yet tested in active outpatient clinical workflow.
Ng et al. [25] China 2022 Experimental Prototype with simulated user study	Real‐time 3D holographic provider ‘teleportation’ via HoloLens 2 using Azure Kinect cameras and Unity	24; 12 novices, 12 experts with >5 years experience in US‐guided procedures.	Time to complete phantom biopsy; Accuracy	AR reduced task time significantly for both novices and experts; tracking showed sub‐mm accuracy	Need for specialised equipment.
Sparwasser et al. [26] Germany 2023 Pilot Study	Magic Leap 1 smart glasses projected MRI‐identified lesion location in real‐time	4 Patients undergoing prostate biopsy with MRI visible lesions	AR‐assisted cancer detection rate	Prostate cancer detection was more likely for AR systems than systematic biopsy (46% to 27%). High feasibility scores. Median execution time: 28 min. Users reported that AR display was non‐intrusive and intuitive	Manual registration required; limited field of view; need for specialised equipment
Di Mitri et al. [17] Italy 2023 Cohort Prospective	Use of HoloLens 2 to visualise 3D anatomical reconstructions from imaging data to assist in complex pediatric surgical planning.	10 pediatric surgeons evaluated the tool across various surgical cases.	Perceived utility, ease of use, realism, anatomical accuracy and value for surgical planning.	3D visualisations improved anatomical clarity compared to 2D CT scans, supporting better surgical planning and risk reduction. They also accelerated learning for complex procedures and enhanced team collaboration and decision‐making	Small sample size, no clinical outcome measures, reliance on preprocessed imaging data; need for specialised equipment

**TABLE 5:** Rehabilitation and functional recovery support.

Author, country, year, study type	AR intervention details	Sample size (n), population characteristics	Outcome measure	Key findings	Limitations
Bank et al. [18] The Netherlands 2018 Observational feasibility study	Three customised AR exercise games used to assess upper extremity motor function; used wearable AR hardware with marker tracking and contactless hand tracking. Hardware used: AIRO II optical see‐through HMD, Leap Motion sensor, Logitech C922 webcam, Microsoft Kinect v2 sensor.	30; 10 patients with Parkinson's Disease; 10 stroke patients with >12 weeks post‐stroke; 10 age‐matched healthy controls	Maximum reach distance; speed, goal‐directedness, hand opening and obstacle avoidance; usability (system usability scale), task load (NASA‐TLX), presence and engagement.	AR games were feasible, engaging and usable; movement differences between groups were detected using AR.	Limited field of view, ceiling/floor effects depending on impairment levels; need for specialised equipment
Yeo et al. [19] South Korea 2021 Protocol for Multi‐Center RCT	Home‐based interactive UINCARE Home_ device with real time motion tracking. Real time motion tracking of 25 joints; Exercise guidance and real‐time performance feedback; Remote monitoring by researchers via platform.	100 patients with stage I or II adhesive capsulitis	Change in passive range of motion (PROM) of the affected shoulder	Not applicable	Not evaluated.
Wang et al. [20] China 2023 Protocol for prospective RCT	Smartphone‐based AR integrity with computer vision; allows for interactive feedback and 3D skeletal tracking.	64 Patients with isolated longitudinal meniscus tears	Improvement in knee function and pain reduction	Not applicable	Not evaluated
Borreson et al. [21] United States of America 2023 Cross‐sectional pilot study	Two Xbox Kinect RGB‐D cameras, Force dimension Omega.3 haptic controller and 3D‐capable television and active 3D glasses used with software for real‐time force and movement tracking.	15 Participant male veterans with upper extremity impairment (average age: 63)	Inter‐rater agreement between remote (ARTESH) and in‐person examinations on PROM and maximum isometric strength (MIS)	ARTESH showed promising agreement with in‐person exams.	Limitations in movement volume and force capability of haptic devices; requires specialised equipment and high‐speed internet.
Lendaro et al. [22] Sweden 2025 RCT Study	Wearable AR and VR device; using surface electrodes, computer displays and virtual imbs with AR markers	81 Participants with phantom limb pain (PLP); adult amputees with chronic PLP	Change in Phantom limb pain (Pain Rating Index, PRI from short‐form mcgill pain questionnaire)	PLP decreased by 64.5% (PME: Overt phantom movement) and 68.2% (PMI: mental imagery of phantom movement); 71%(PME) and 68%(PMI) achieved clinically meaningful pain reduction (>50%).	Therapists had to undergo training; minimal learning curve; need for specialised equipment.

Additionally, there may be systemic barriers to implementation. There is a risk of clinical or physician reluctance, as some may view AR as burdensome, especially if training is required or if integration with pre existing workflows is difficult. There is also the matter of patient comfort with technology–especially among older populations or those with limited digital literacy.

Even so, given the right conditions, AR can lead to immense improvements in the outpatient workflow. Not only has AR been shown to have high usability scores, but its efficacy has also been proved with consistent user satisfaction and recommendation rates [9, 10, 11, 13, 17, 25]. Furthermore, the ability to provide 3D visualisations vastly increases patient understanding [9, 10, 11]. AR also has the potential to enhance remote monitoring and home‐based therapy [19, 22, 27].

Based on our findings, we can arrange AR applications by their readiness for clinical implementation. For the present study, the brainstorming was done amongst four authors (AK, AS, FT and LE) to determine the gap between the current application status and routine clinical usage in the future based on limitations of studies included in this review. Applications that are ready for implementation include phantom limb pain treatment, patient education for complex procedures and medical device use [9, 10, 11, 14, 15, 24, 27]. Applications that still require refinement include using AR as a cognitive assessment tool, using AR for low‐risk procedural guidance, and using AR for home‐based rehabilitation [19, 20, 21, 22, 27]. Finally, applications that require major development include high‐risk surgical navigation, diagnostic applications requiring high reliability and fully autonomous AR‐guided procedures.

This review is not without limitations. Foremost, all the studies included in the review have relatively small sample sizes–with the largest having 121 participants–limiting generalizability [12]. Also, there are issues with the assortment of study types. There is a predominance of pilot studies–as this is a relatively new frontier–limiting the strength of the evidence, and several studies reported protocols without outcome data [10, 13, 14, 15, 16, 17, 23, 22]. Furthermore, there is some heterogeneity of interventions and outcomes in included studies.

Most of the studies that included direct patient contact with AR interventions frequently referenced consent and compliance with HIPAA guidelines and IRB protocols. Privacy safeguards in AR systems included anonymised patient data and a secure AR interface. This is important given that AR systems often involve sensitive patient information. For AR to be further adopted in outpatient settings, ethics and patient privacy must be ensured with strict compliance to HIPAA guidelines.

Based on our study, there are many areas for future research regarding AR in outpatient care. Most importantly, there is a necessity for large‐scale RCTs with standardised outcome measures. There is also a need for platform standardisation studies to ensure cross‐device reliability of AR systems; long‐term follow‐up studies to assess long‐term sustained benefits of AR systems; cost‐effectiveness analyses to guide implementational decisions; and studies developing clinical guidelines for AR implementation.

## 5 Conclusion

Augmented reality has been proved to have the potential to transform outpatient care across five main areas: patient education, cognitive and functional assessment, medical device interaction, procedural guidance and rehabilitation. Studies over the past 10 years consistently support that AR enhances patient comprehension, engagement and procedural accuracy while allowing for remote monitoring and personalised therapy. Furthermore, AR interventions demonstrate high usability and clinical relevance. Nevertheless, limitations such as hardware complexity and inconsistent technical performance remain. Overall, AR demonstrates great potential in improving outpatient workflows and outcomes and allowing for personalised care.

## Author Contributions

Study conception and design: Archan Khandekar and Dipen Parekh. Acquisition of data: Aryan Shah, Archan Khandekar, Luis A Esparza Miranda, Frida Toscano Bello, Bruno Liebl, Jonathan Ryan and Pedro Angelo Basei de Paula. Statistical analysis: Ansh Bhatia and Timothy Guerard. Interpretation of statistical analysis: Aryan Shah, Archan Khandekar, Luis A Esparza Miranda, Frida Toscano Bello, Bruno Liebl, Jonathan Ryan and Pedro Angelo Basei de Paula. Drafting the article: Aryan Shah and Archan Khandekar. Revision: Aryan Shah, Archan Khandekar, Luis A Esparza Miranda, Frida Toscano Bello, Bruno Liebl, Jonathan Ryan, Pedro Angelo Basei de Paula and Dipen J. Parekh. Final approval of version to be submitted: Aryan Shah, Archan Khandekar, Luis A Esparza Miranda, Frida Toscano Bello, Bruno Liebl, Jonathan Ryan, Pedro Angelo Basei de Paula and Dipen J. Parekh. Contributed to data acquisition, interpretation of statistical analyses, and manuscript drafting and revision. Participated in the critical review of intellectual content and approved the final version of the manuscript for submission: Luis A. Esparza Miranda.

## Funding

The authors have nothing to report.

## Conflicts of Interest

The authors declare no conflicts of interest.

## Data Availability

The data files included in this article could be shared upon request.
